# Effect of non-invasive ventilation and high-flow nasal cannula on hospital mortality in COVID-19-induced acute respiratory failure: a meta-analysis

**DOI:** 10.31744/einstein_journal/2026RW0695

**Published:** 2025-12-01

**Authors:** Jakson Henrique Silva, Anna Luísa Araújo Brito, Redha Taiar, Bruno Amorim Moraes, Anderson Brasil Xavier, Wagner Souza Leite, Maria das Graças Rodrigues de Araújo, Daniella Cunha Brandão, Armele de Fátima Dornelas Andrade, Shirley Lima Campos

**Affiliations:** 1 Department of Physical Therapy Health Sciences Center Universidade Federal de Pernambuco Recife PE Brazil Department of Physical Therapy, Health Sciences Center, Universidade Federal de Pernambuco, Recife, PE, Brazil.; 2 Department d’Education Physique et Sportive Université de Reims Champagne-Ardenne France Department d’Education Physique et Sportive, Université de Reims Champagne-Ardenne, France.; 3 Department of Medicine Universidade de Pernambuco Recife PE Brazil Department of Medicine, Universidade de Pernambuco, Recife, PE, Brazil.

**Keywords:** COVID-19, SARS-CoV-2, Oxygen inhalation therapy, Noninvasive ventilation, Intubation, Mortality, Respiratory insufficiency

## Abstract

**Background:**

Non-invasive respiratory support strategies, such as high-flow nasal cannula therapy and non-invasive ventilation, were widely employed during the coronavirus disease 2019 (COVID-19) pandemic, yet their comparative effectiveness remains uncertain.

**Objective:**

To compare the effects of high-flow nasal cannula therapy, non-invasive ventilation, and conventional oxygen therapy on intubation rates and hospital mortality in adults with COVID-19-related acute respiratory failure.

**Methods:**

A systematic review and meta-analysis was conducted following PRISMA and Cochrane guidelines, with searches performed in nine databases for publications up to May 2023. Eligible studies were those on adults (≥18 years) with confirmed severe acute respiratory syndrome coronavirus 2 infection and that included intubation and mortality as primary outcomes. Risk of bias was assessed using the National Institutes of Health Quality Assessment Tool for Observational Cohorts and the Cochrane Risk of Bias tool. Pooled results were reported as odds ratios (ORs) with 95% confidence intervals (95%CIs).

**Results:**

Forty-one studies were included in the review and ten in the meta-analysis (2,843 patients). High-flow nasal cannula therapy did not differ from non-invasive ventilation in terms of the intubation rate (OR=1.07, 95%CI=0.89-1.29, p=0.45) but was superior to oxygen therapy (OR=0.79, 95%CI=0.64-0.97, p=0.02). High-flow nasal cannula therapy was also associated with lower mortality than non-invasive ventilation (OR=0.62, 95%CI=0.51-0.76, p<0.0001) but did not differ from oxygen therapy (OR=1.06, 95%CI=0.84-1.33, p=0.64). Substantial heterogeneity was observed in the subgroup analyses (I^2^=64%-90%).

**Interpretation:**

High-flow nasal cannula therapy may reduce the need for intubation compared with oxygen therapy and may lower the hospital mortality rate compared with non-invasive ventilation. However, heterogeneity in the studies suggests that patient-specific factors and disease severity may influence outcomes.

**Conclusion:**

High-flow nasal cannula therapy shows potential benefits over oxygen therapy and non-invasive ventilation for COVID-19-related acute respiratory failure, particularly in the mortality rate. Clinical use of these therapies should be context-specific, given the need for cautious interpretation of our results and for further high-quality trials.

Prospero database registration: ID CRD 42020226936.

## ❚INTRODUCTION

Coronavirus disease 2019 (COVID-19) has a range of clinical manifestations, from mild to severe conditions. Severe COVID-19 frequently progresses to acute hypoxemic respiratory failure, which necessitates the provision of high levels of oxygen and invasive mechanical ventilation (IMV).^( 1 , 2 )^ Early studies indicated that more than 60% of patients required intubation within 24 h of hospital admission and approximately 80% during their stay in the intensive care unit.^( 3 )^

The high mortality among patients with severe COVID-19 who were receiving IMV created a substantial burden on healthcare systems, leading to a search for efficient and safe therapies for COVID-19 in the acute phase.^( 3 )^ Some studies on non-invasive ventilation strategies in this population have shown positive outcomes, including a reduction in the need for IMV and a consequent decrease in mortality rates compared with those of other therapies.^( 3 - 5 )^

A recent systematic review of nine studies (one randomized controlled trial [RCT], seven retrospective studies, and one prospective study; 1,582 participants) revealed no significant difference between high-flow nasal cannula therapy (HFNC) and non-invasive ventilation (NIV) in the reduction of escalation to IMV.^( 6 )^ Neither the incidence of IMV nor the number of deaths (without a time limitation) differed between the groups.^( 6 )^

Considering the continued emergence of new severe acute respiratory syndrome coronavirus 2 (SARS-CoV-2) variants and the potential for new outbreaks, synthesized data on the efficacy of NIV and HFNC concerning mortality and intubation rates are needed.

## OBJECTIVE

That was the aim of our meta-analysis, focusing on adults with COVID-19-associated acute respiratory failure.

## METHODS

This systematic review was registered with the International Prospective Register of Systematic Reviews. It adhered to the principles of both the Cochrane^( 7 )^and the Preferred Reporting Items for Systematic Review and Meta-Analyses (PRISMA)^( 8 )^ guidelines.

### Search strategy

The detailed search strategy is provided in Table 1S , Supplementary Material. The search was conducted for studies published until May 2023, without restrictions on language or year of publication.

**Table 1S:** Database search strategy

Data base	
Cochrane Central Register of Controlled Trials (CENTRAL) n=343	(COVID-19 OR coronavirus OR Sars-CoV-2) AND (((Noninvasive OR “Non invasive”) AND (Respiratory OR Ventilatory OR Ventilation)) OR “Non-invasive mechanical ventilation” OR “Continuous Positive” OR “Continuous positive airway pressure” OR “Bilevel positive airway pressure” OR “High Flow Nasal” OR “High flow nasal cannula” OR “High flow nasal oxygen”)
ClinicalTrials.gov n=29	COVID OR coronavirus OR Sars-CoV-2 | ((Noninvasive OR “Non invasive”) AND (Ventilation OR Ventilatory OR Respiratory)) OR “Non-invasive mechanical ventilation” OR “Continuous Positive” OR “Continuous positive airway pressure” OR “Bilevel positive airway pressure” OR “High Flow Nasal” OR “High flow nasal cannula” OR “High flow nasal oxygen”
SciVerse Scopus n=291	(TITLE-ABS-KEY (( covid-19 OR coronavirus OR sars-cov-2) AND ( “Noninvasive Ventilat*” OR “Non-invasive Ventilat*” OR niv OR “Continuous Positive” OR “Continuous positive airway pressure” OR “Bilevel positive airway pressure” OR “High Flow Nasal” OR “High flow nasal cannula” OR “High flow nasal oxygen” ) ) AND TITLE ( ( covid OR coronavirus OR sars-cov-2 ) AND ( noninvasive OR ventilat* OR respirat* OR niv OR “Continuous Positive” OR “CPAP” OR “BIPAP” OR “High Flow Nasal” OR “HFNC” OR “HFNO” ) ) )
ScienceDirect n=117	(COVID OR Coronavirus OR Sars-CoV-2) AND (“Noninvasive Ventilation” OR “Non-invasive Ventilation” OR “Non-invasive mechanical ventilation” OR “High Flow Nasal” OR “High flow nasal cannula” )
Google Scholar n=200	(COVID-19 OR coronavirus OR Sars-CoV-2) AND (((Noninvasive OR “Non invasive”) AND (Respiratory OR Ventilatory OR Ventilation)) OR “Non-invasive mechanical ventilation” OR “Continuous Positive” OR “Continuous positive airway pressure” OR “Bilevel positive airway pressure” OR “High Flow Nasal” OR “High flow nasal cannula”)
Scientific Electronic Library Online (SciELO) n=2	(ti:(((covid OR coronavirus OR sars-cov-2 ) AND ( noninvasive OR ventilation OR ventilatory OR respiratory OR niv OR “Continuous Positive” OR cpap OR bipap OR “High Flow Nasal” OR hfnc OR hfno OR “Ventilação Não Invasiva” OR VNI OR “Cânula Nasal” OR CNAF)))) AND (ab:((“Noninvasive Ventilation” OR “Non invasive Ventilation” OR “Non-Invasive Ventilation” OR NIV OR “Continuous Positive” OR “Continuous positive airway pressure” OR “Bilevel positive airway pressure” OR “High Flow Nasal” OR “High flow nasal cannula” OR “High flow nasal oxygen” OR “Ventilação Não Invasiva” OR “Ventilação não invasiva” OR “Cânula Nasal” OR “Canula nasal de alto fluxo”)))
Latin American and Caribbean Health Sciences Literature (LILACS) n=15	((COVID OR “Infecções por Coronavirus”) AND (“Ventilação Não Invasiva” OR VNI OR “Cânula Nasal” OR “Cânula nasal de alto fluxo” OR CPAP OR BIPAP OR “Pressão Positiva Contínua”)) OR ((COVID OR Coronavirus OR Sars-CoV-2) AND (“Noninvasive Ventilation” OR “Non-invasive Ventilation” OR NIV OR “High Flow Nasal” OR HFNC))
MEDLINE via Pubmed n=243	(((covid[Title] OR coronavirus[Title] OR sars-cov-2[Title]) AND (noninvasive[Title] OR “non-invasive”[Title] OR “non invasive”[Title] OR ventilat*[Title] OR respirat*[Title] OR niv[Title] OR “Continuous Positive”[Title] OR “Continuous positive airway pressure”[Title] OR “Bilevel positive airway pressure”[Title] OR “High Flow Nasal”[Title] OR “High flow nasal cannula”[Title] OR “High flow nasal oxygen”[Title])) AND ((covid-19[Title/Abstract] OR coronavirus[Title/Abstract] OR sars-cov-2[Title/Abstract]) AND ( “Noninvasive Ventilat*”[Title/Abstract] OR “Non invasive Ventilat*”[Title/Abstract] OR “Noninvasive Respirat*”[Title/Abstract] OR “Non invasive Respirat*”[Title/Abstract] OR niv[Title/Abstract] OR “Continuous Positive”[Title/Abstract] OR cpap[Title/Abstract] OR bipap[Title/Abstract] OR “High Flow Nasal”[Title/Abstract] OR hfnc[Title/Abstract] OR hfno[Title/Abstract])))
medRxiv n=703	(COVID OR Coronavirus OR Sars-CoV-2) AND (“Noninvasive Ventilation” OR NIV OR “Non-Invasive Ventilation” OR “High Flow Nasal” OR HFNC)

In the title, abstract, and keywords of articles, we queried for “COVID-19,” “noninvasive ventilation,” and “high flow nasal cannula,” along with their respective variants, abbreviations, and combinations in both English and Brazilian Portuguese. These terms included “SARS-CoV-2,” “coronavirus,” “oxygen therapy,” “non-invasive ventilation,” “NIV,” “respiratory,” “ventilatory,” “ventilation,” “continuous positive,” “continuous positive airway pressure,” “CPAP,” “bilevel positive airway pressure,” “BIPAP,” “HFNC,” “high flow nasal oxygen,” and “HFNO.” To optimize the search, we used the Boolean logical operators “AND” and “OR” to combine terms that were indexed to DeCS/MeSH descriptors.

This search was executed in the following electronic databases: Cochrane Central Register of Controlled Trials (CENTRAL), ClinicalTrials.gov, SciVerse Scopus, ScienceDirect, Google Scholar, Scientific Electronic Library Online (SciELO), Latin American and Caribbean Health Sciences Literature (LILACS), MEDLINE via PubMed, and medRxiv.

### Eligibility criteria

This systematic review included studies involving adults (aged 18 years and above) with acute respiratory failure due to a confirmed SARS-CoV-2 infection. Eligible study designs were randomized and non-randomized clinical trials, observational cohort studies (prospective or retrospective), case series, case-control studies, and case reports.

Studies that did not include outcomes related to intubation or hospital mortality associated with NIV (administered through nasal, facial, or helmet interfaces in continuous or bilevel positive airway pressure modes), HFNC, and conventional oxygen therapy were excluded. Additionally, studies focused primarily on interventions aimed at weaning from IMV, and those conducted in settings where these interventions were not the primary approach to ventilatory support, were excluded. Studies focused exclusively on the risk of nosocomial transmission without outcomes related to respiratory support were also excluded.

### Screening and data extraction

Titles and abstracts were screened using the Rayyan online software^( 9 )^ by two independent reviewers. The full text of the selected studies was then screened, and in cases of disagreement between the reviewers, a third evaluator was consulted for the inclusion decision.

The extracted data were the authors, year, country, study design, sample characteristics, comorbidities, medications used, intervention and its parameters, primary outcomes (intubation and mortality), and secondary outcomes. The secondary outcomes were a) oxygenation and ventilation, as determined via arterial blood gas parameters (the partial pressure of oxygen in the arterial blood [PaO_2_], the PaO_2_/fraction of inspired oxygen [FiO_2_] ratio, and the partial pressure of carbon dioxide in the arterial blood); b) the PaO_2_/FiO_2_ ratio category (mild, moderate, and severe) according to the Berlin definition;^( 10 )^ and c) self-reported respiratory health symptoms (flu, common cold, cough, and runny nose) or those measured using a visual assessment scale.

### Quality assessment

Study heterogeneity was examined in relation to study designs. Observational studies were assessed using the National Institutes of Health (NIH, USA) Quality Assessment Tool for Observational Cohort and Cross-Sectional Studies (https://www.nhlbi.nih.gov/node/80102). Randomized controlled trials were assessed using the Cochrane Risk of Bias Tool for Randomized Trials (RoB2) across six domains, classifying the risk as “low,” “High,” or “some concerns.”^( 7 )^

### Data analysis

Descriptive analyses were applied to non-comparative studies to examine therapy modalities and protocols, including their associations with different body positions. Meta-analyses of the primary outcomes, orotracheal intubation, and mortality rate were performed by grouping studies into subsets of therapy modalities (HFNV, NIV, or oxygen therapy) to mitigate heterogeneity.

The Mantel-Haenszel (M-H) odds ratio (OR) was calculated using a random-effects model with a 95% confidence interval (95%CI). Heterogeneity was assessed with the I^2^ statistic. Data were extracted and analyzed using RevMan software (version 5.4.1; The Cochrane Collaboration, Copenhagen, Denmark). These rigorous methodologies ensured the robustness of the synthesized results and accounted for potential biases arising from missing data and reporting biases.

## RESULTS

**Figure 1:**
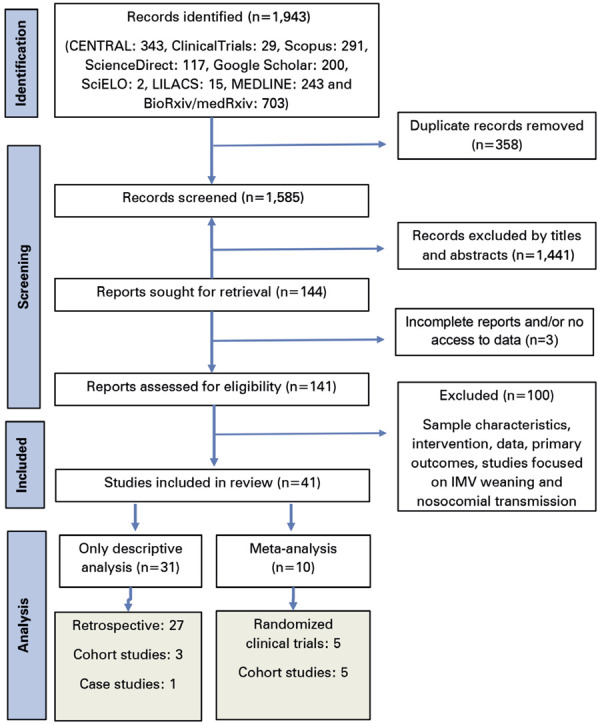
Flow diagram of study selection

A total of 1,943 studies were initially identified, of which 41 were included in the final analysis ( Figure 1 ). Ten were included in the meta-analysis, comprising five RCTs^( 11 - 15 )^ and five cohort studies.^( 16 - 20 )^

The remaining 31 studies were excluded from the meta-analysis owing to the absence of comparative or control groups, lack of data, or changes in body positioning ( Table 2S , Supplementary Material).

**Table 2S:** Characteristics of the thirty one studies eligible for descriptive analysis

Author, Year, Country	Intervention	Full Sample (N)/Sample per group	Age (years, yo) - total/ per group	Sex (M/F)	Body mass index (BMI)	Comorbidities	Medication	Intervention parameters	Primary outcomes	Secondary outcomes	Other results
Cohort											
Ferrando et al., 2020 ^(^ ^32^ ^)^ Spain	G1 : HFNC G2 : HFNC + Prone position	N=199 G1 : 144 G2 : 55	61.5 years old G1 : 63 G2 : 60 (median)	G1 : 105/39 G2 : 42/13	G1 : 27.3 G2 : 26.8	G1 : SAH (41.7%), DM (16%), Obesity (20.8), CKD (9.7%), Dyslipidemia (10.4) and COPD: (4.2%) G2 : SAH (6.4%), DM (16.4%), COPD (7.3%) and CHF (3.6%)	G1 : Corticosteroid: 9 (6.3%), Bronchodilator: 35 (24.3%) G2 : Corticosteroid: 2 (3.6%), Bronchodilator: 10 (18.2%)	Not reported	G1 : Intubation rate: 60 (41.7%) ICU mortality**: 17/122 (13.9%) G2 : Intubation rate: 22 (40%) ICU mortality**: 8/49 (16.3%) **Excluding patients who are in ICU.	G1 : PaCO_2_: 39.9 mmHg PaO_2_/FiO_2_: 92.5 mmHg G2 : PaCO_2_: 41.2 mmHg PaO_2_/FiO_2_: 103 mmHg	G1 : ICU length of stay**: 7.5 [4-14] G2 : ICU length of stay**: 8 [5-14] **Excluding patients who are in ICU.
Ramirez et al., 2020^(^ ^33^ ^)^ Italy	CPAP + Prone position	N=90	61 years old	72/18	Not reported	SAH: (33%), DM (19%), Pneumopathies: (6%) and CHF (2%)	Not reported	Not reported	Intubation rate: 32 (38%) Mortality: 17 (19%)	Not reported	Not reported
Mellado et al., 2021^(^ ^34^ ^)^ Spain	G1 : Early intubation G2 : HFNC	N=122 G1 : 61 G2 : 61	61.5 years old G1 : 61 (11) G2 : 62 (11) mean (SD)	G1 : 25/36 G2 : 34/27	G1 : 28.8 (4.3) G2 : 28.5 (5.5)	G1 : Immunosuppression 2 (3.3) G2 : Immunosuppression: 4 (6.6) and Active cancer: 6 (9.8)	G1 : Steroids: 47 (77%) G2 : Steroids: 45 (73.8%)	Not reported	G1 : Intubation rate: 61 (100%) Mortality: 13 (21%) G2 : Intubation rate: 23 (38%) Mortality: 9 (15%)	G1 : PaCO_2_, mmHg - mean (SD): 37 (8) PaO_2_/FiO_2_ mean (SD): 117(51) mmHg G2 : PaCO_2_, mmHg - mean (SD): 38 (12) PaO_2_/FiO_2_ mean (SD): 121(48) mmHg	G1 : Length of stay in ICU (mean): 20 days. SOFA score—median [IQR]: 5 [3-7] G2 : Length of stay in ICU (mean): 11 days SOFA score-median [IQR]: 4 [4-7]
Retrospective											
McDonough et al., 2020 ^(^ ^35^ ^)^ United States of America	G1 : HFNC G2 : NIV (face mask)	N=83 G1 : 54 G2 : 29	66 years old G1 : 65 (56-78) G2 : 67 (53-75)	G1 : 33/21 G2 : 20/9	G1 : 28.4 G2 : 30.4 (26.3 - 34)	G1 : SAH: (59%), DM: (56%), CHF: (15%), COPD/Asthma: (15%) G1 : SAH: (62%), DM: (48%), CHF: (35%) and COPD/Asthma : (28%)	All: Inhaled pulmonary vasodilator: 9 (10.8%)	Not reported	G1 : Intubation rate:36 (67%) Mortality: 17 (31%) G2 : Intubation rate: 22 (76%) Mortality: 11 (38%)	G1 : PaO_2_/FiO_2_ mean (IQR): 157mmHg (106-224) G2 : PaO_2_/FiO_2_ mean (IQR): 107mmHg (84-183.5)	G1 : Length of hospital stay: 14.5 ICU stay: 8 G2 : Length of hospital stay: 19.5 ICU stay: 11.4
Demoule et al., 2020 ^(^ ^36^ ^)^ France	CG: Standard treatment (without HFNC) EG: HFNC	N=379 CG: 233 EG: 146	61.5 years old CG: 63 EG: 60	CG: 174/59 EG: 116/30	CG: 28 EG: 27	CG: SAH: (52%), DM: (31%), COPD (6%) and Asthma: (5%) - CHF: (10%) EG: SAH: (46%), DM: (29%), COPD (5%) and Asthma: (5%) - CHF: (7%)	Not reported	Not reported	CG: Intubation rate: 172 (74%) Mortality: 70 (30%) EG: Intubation rate: 82 (56%) Mortality: 31 (21%)	CG: PaO_2_/FiO_2:_ 130 (97-195) EG: PaO_2_/FiO_2: _130 (97-195)	Not reported
Alharthy et al., 2020 ^(^ ^37^ ^)^ Saudi Arabia	G1 : CPAP - Helmet G2 : HFNC	N=30 G1 : 15 G2 : 15	45 years old G1 : 46 (38-55) G2 : 44 (37-57)	G1 : 3/12 G2 : 13/2	G1 : 24 (20-29) G2 : 24 (20-29)	Not reported	Not reported	Not reported	G1 : Intubation rate: 3 (20%) G2 : Intubation rate: 2 (13%)	G1 : PaO_2_/FiO_2_: Before: 211 (198-235)/After: 377 (344-433) G2 : PaO_2_/FiO_2_: Before: 213 (199-241)/After 380 (352-421)	Not reported
**Author, Year, Country**	**Intervention**	**Full Sample (N)/Sample per group**	**Age (years, yo) - total/ per group**	**Sex** **(M/F)**	**Body mass index (BMI)**	**Comorbidities**	**Medication**	**Intervention parameters**	**Primary outcomes**	**Secondary outcomes**	**Other results**
Gaulton et al., 2020 ^(^ ^38^ ^)^ United States of America	G1 : CPAP - Helmet G2 : HFNC	N=59 G1 : 17 G2 : 42	58.5 years old G1 : 56±15 G2 : 61±16	G1 : 14/3 G2 : 14/28	G1 : 34.8 (± 7.6) G2 : 35.8±9	G1 : SAH: 11 (64.7%), DM: 8 (47.1%) andCKD: 4 (23.5%) G2 : SAH: 24 (57.1%), DM: 13 (31%) and CKD: 13 (31%)	Not reported	Not reported	G1 : Intubation rate: 3 (17.7%) Mortality: 1 (5.9%) G2 : Intubation rate: 22 (52.4%) Mortality: 34 (81%)	Not reported	Not reported
Duan et al., 2021 ^(^ ^27^ ^)^ China	G1 : HFNC G2 : NIV	N=36 G1 : 23 G2 : 13	57.5 years old G1 : 65 G2 : 50	G1 : 12/11 G2 : 12/1	Not reported	G1 : SAH: (26%), DM (17%) and Chronic Heart Disease (17%) - Chronic Pneumopathy (4%) G2 : SAH: (23%)	Not reported	G1 : Flow (30-60L/min), FiO_2_ (SpO_2_ >93%) and temperature (31-37°C). + G2 : Initial NIV CPAP 4cmH_2_O. then BIPAP 8-10cmH_2_O	G1 : Intubation rate: 4 (17%) Mortality: 1 (4%) G2 : Intubation rate:2 (15%) Mortality: 1 (8%)	G1 : PaO_2_/FiO_2_: 196±46 mmHg (admission) PaCO_2_: 36±5 (after 24 hours of therapy) PaO_2_/FiO_2_: 224±92 (after 24 hours) G2 : PaO_2_/FiO_2_: 165±48 (at admission) PaCO_2_: 36±5 (after 24 hours of therapy) PaO_2_/FiO_2_: 202±65 (after 24 hours)	G1 : SOFA: 4±2 G2 : SOFA: 4±1
Sayan et al., 2021 ^(^ ^22^ ^)^ Turkiye	CG: Reservoir mask EG: HFNC	N=43 CG: 19 EG: 24	66 years old CG: 69±12.3 EG: 63±12	CG: 13/6 EG: 18/6	CG: 26.5 EG: 26.5	CG: SAH: (63.2%), DM: (26.3%), Coronary disease (15.8%) and COPD (0%) EG: SAH: (25%), DM: (12.5%), CAD (8.3%) and COPD (8.3%)	Not reported	CG: Reservoir mask with flow 6-15L/min. SpO_2_ >93%. FiO_2_ (%) 21 + 4 flow emL/min. EG: Flow 30-60L/min, FiO_2_ 40-90%. SpO_2_ target >93%. Temperature 31-37ºC. Continuous application at the beginning and intermittently after PaO_2_/FiO_2_ >250 mmHg and clear clinical improvement.	CG: Intubation rate: 16 (84%) Mortality: 16 (84%) EG: Intubation rate: 13 (54%) Mortality:12 (50%)	CG: PaO_2_: 68.1±18.3 (39-120) PaCO_2_: 38.9±17.3 mmHg (21-99) PaO_2_/FiO_2_: 184.2±49.5 mmHg (105.4±324.3) EG: PaO_2_: 81.7±26.7 mmHg (54-169) PaCO_2_: 34.2±7.2 mmHg (22-57) PaO_2_/FiO_2_: 198.5±51.3 mmHg (135-382) (Mean±SD min-max) Values after 24 hours of therapy	CG: Length of ICU stay: 9±7.9 days EG: Length of ICU stay:: 9.8±4.8 days
Bonnet et al., 2021 ^(^ ^28^ ^)^ France	G1 : Oxygen therapy G2 : HFNC	N=138 G1 : 62 G2 : 76	60 years old G1 : 60 (51-67) G2 : 60 (52-67)	G1 : 50/12 G2 : 62/14	G1 : 27 (26-33) G2 : 29 (25-33)	G1 : Immunosuppression: 9 (14%), SAH: 19 (31%), DM: 19 (31%), IRpC: 2 (3%) and CKF: 3 (5%) G2 : Immunosuppression: 11 (14%), SAH: 37 (49%), DM: 24 (42%), IRpC: 2 (3%) and CKF: 4 (5%)	G1 : Steroids: 66 (48%) G2 : Steroids: 25 (40%)	G1 : Non-Rebreathing Facial Mask with 6L/min or more. (flow rate adjusted to maintain a SpO_2_ higher than 92%). G2 : Flow: 60L/min is FiO_2_: 100% (adjusted to keep SpO_2_ higher than 92%).	G1 : Intubation rate: 46 (74%) Mortality rate: 15 (24%) (on day 28) and 16 (26%) (on day 60) G2 : Intubation rate: 39 (51%) Mortality rate: 9 (12%) (on day 28) and 12 (16%) (on day 60)	G1 : PaO_2_ median. mmHg (IQR): 71 (62-85) Respiratory rate, median (IQR): 30 (26-35) G2 : PaO_2_ median. mmHg (IQR): 69 (63-82) Respiratory rate, median (IQR): 33 (28-36)	G1 : Average length of ICU stay. days (IQR):12.5 (4-24) G2 : Average length of ICU stay. days (IQR): 11 (5-20)
**Author, Year, Country**	**Intervention**	**Full Sample (N)/Sample per group**	**Age (years, yo) - total/ per group**	**Sex** **(M/F)**	**Body mass index (BMI)**	**Comorbidities**	**Medication**	**Intervention parameters**	**Primary outcomes**	**Secondary outcomes**	**Other results**
Jarou et al., 2021 ^(^ ^39^ ^)^ United States of America	G1 : No HFNC G2 : HFNC	N=123 G1 : 28 G2 : 95	71.85 years old G1 : 69 G2 : 65	G1 : 16/12 G2 : 48/47	G1 : 31.9 G2 : 30.8 (median)	G1 : CKD: 12 (42.9%), COPD: 7 (25%), DM: 12 (53.6%) -, SAH: 20 (71.4%) and AMI:6 (21.4%) G2 : CKD: 43 (45.3%), COPD: 27 (28.4%), DM: 45 (47.4%), SAH: 83 (87.4%) and AMI: 22 (23.2%)	Not reported	G1 : Nasal cannula with flow ≥6L/min G2 : Flow of 40 a 60L/min adjusted according to the work of breathing; FiO_2_: 100% adjusted to maintain a saturation between 92 e 96%	G1 : Intubation rate: 24 (85.7%) Mortality rate: 14 (50%) G2 Intubation rate: 31 (32.6%) Mortality rate: 30 (31.6%)	Not reported	G1 : Length of hospital stay (median): 8.6 G2 : Length of hospital stay (median): 6.0
Kabak et al., 2021^(^ ^40^ ^)^ Turkiye	G1 : HFNC G2 : Non-respiratory Masks + Nasal Cannula	N=54 G1 : 26 G2 : 28	64.79 years old G1 : 64.30±15.73 G2 : 65.28±13.32	G1 : 19/7 G2 : 17/11	Not reported	G2 : SAH: 16 (61.5%), DM: 3 (11.5%), CAD: 3 (11.5%), - COPD: 1 (3.8%), Asthma: 0 (0%), CHF: 2 (7.7%) and CKD: 1 (3.8%) G2 : SAH: 16 (57.1%), DM: 7 (25%), CAD: 2 (7.1%), COPD: 4 (14.3%), Asthma: 2 (7.1%), CHF: 0 (0%) and CKD: (3.8%)	Not reported	Not reported	G1 : Mortality rate: 9 (34.6%) G2 : Mortality rate: 7 (25%)	G1 : PaO_2_/FiO_2_: 189.19±24.38 mmHg G2 : PaO_2_/FiO_2_: 189.64±28.88 mmHg	G1 : Length of stay in the ICU (days): 10.19±6.29 Hospital Stay: 17.88±8.28 G2 : Length of stay in the ICU (days): 8.7±5.2 Hospital stay: 23.60±12.92
Zhao et al., 2021 ^(^ ^41^ ^)^ China	G1 : NIV G2 : HFNC	N=41 G1 : 24 G2 : 17	66 years old G1 : 68 (62-76) G2 : 64 (54-74)	G1 : 18/6 G2 : 10/7	Not reported	G1 : DM: 7 (29.2%), SAH: 14 (58.3%), CAD: 1 (4.2%), COPD: 2 (8.3%) and cerebrovascular disease: 2 (8.3%) G2 : DM: 2 (11.8%), SAH: 6 (35.3%), CAD: 2 (11.8%), COPD: 2 (11.8%) and cerebrovascular disease: 2 (11.8%)	Not reported	Not reported	G1 : Intubation rate: (37.5%) Mortality: 4 (16.7%) G2 : Intubation rate: 12 (70.6%) Mortality: 5 (29.4%)	G1 : PaO_2_/FiO_2_:118.3 (110.15-131.15) G2 : PaO_2_/FiO_2_: 131.15 (111.11-156.31)	Not reported
Rodrigues Santos et al., 2021^(^ ^42^ ^)^ Portugal	G1 : HFNC G2 : CPAP	N=190 G1 : 139 G2 : 51	72 years old G1 : 74.5±19 G2 : 69.6±10.2	G1 : 95/44 G2 : 35/16	G1 : 28.2 (± 5.7) G2 : 29.5±6.2	G1 : SAH: 87 (62.8%), DM: 47 (33.8%), HF: 19 (13.7), CKD: 8 (5.8) and COPD: 8 (5.8%) G2 : SAH: 40 (78.4), DM: 18 (35.3%), HF: 11 (21.6%), CKD: 4 (7.8%) and COPD: 3 (5.9%)	G1 : Steroids: 134 (96.4%) and Rendesivir: 28 (20.1%) G2 : Steroids: 51 (100%) and Rendesivir: 1 (2%)	Not reported	G1 : Intubation rate: 23 (16.54%) Mortality: 38 (27.3%) G2 : Intubation rate: 8 (15.68%) Mortality: 31 (60.8%)	G1 : PaO_2_/FiO_2_: 108.9 (76.1) G2 : PaO_2_/FiO_2_: 70.9 (18.1)	Not reported
**Author, Year, Country**	**Intervention**	**Full Sample (N)/Sample per group**	**Age (years, yo) - total/ per group**	**Sex** **(M/F)**	**Body mass index (BMI)**	**Comorbidities**	**Medication**	**Intervention parameters**	**Primary outcomes**	**Secondary outcomes**	**Other results**
Shoukri et al., 2021^(^ ^43^ ^)^ Saudi Arabia	G1 : HFNC G2 : NIV	N=63 G1 : 37 G2 : 26	66 years old G1 : 67.94±7.83 G2 : 64.10±9.81	G1 : 23/14 G2 : 17/9	Not reported	G1 : SAH: 10 (27.01%), DM: 12 (32.43%), CAD: 2 (5.4%), COPD: 3 (8.10%) and Others: 1 (2.7%) G2 : SAH: 9 (34.61%), DM: 9 (34.61%), CAD: 1 (3.84%), COPD: 3 (11.53%) and Others: 3 (11.53%)	Not reported	Not reported	G1 : Intubation rate: 4 (10.81%) Mortality: 1 (2.7%) G2 : Intubation rate: 4 (11.53%) Mortality: 1 (3.8%)	G1 : PaCO_2_ : 34,67±3.69 (before) PaO_2_/FiO_2_: 191.08±37.83 (before) PaCO_2_ : 38.32±4.32 (after) PaO_2_/FiO_2_: 225.67±44.33 (after) G2 : PaCO_2_ : 35.03±3.99 (before) PaO_2_/FiO_2_: 190.38±42.47 (before) PaCO_2_ : 38.15±3.72 (after) PaO_2_/FiO_2_: 241±49.43 (after)	G1 : SOFA 3.02±0.94 APACHE: 9.78±3.18 G2 : SOFA 2.96±0.77 APACHE: 10.96±3.15
Costa et al., 2022 ^(^ ^44^ ^)^ Brazil	G1 : NIV G2 : HFNC	N=37 G1 : 14 G2 : 23	69.9 years old G1 : 74.5±19 G2 : 65.3±17.7	G1 : 5/9 G2 : 21/2	G1 : 34.4 (± 9.7%) G2 : 29.4±5.5	G1 : SAH: 10 (71.4%), DM: 5 (35.7%), CD 5 (35.7%) and COPD 5 (35.7%) G2 : SAH: 14 (60.8%), DM: 9 (39.1%), CD 5 (21.4%) and COPD 4 (17.4%)	G1 : Hydrocortisone: 6 (42.9%) G2 : Hydrocortisone: 20 (87%)	G1 : PEEP: 9.3±3, PS: 8.2±4.8 and FiO_2:_ 38.2±20.8 G2 : Flow: 45.2±6.5 and FiO_2:_ 52±17.2	G1 : Intubation rate: 16 (69.9%) Mortality 30 days: 3 (8.7%) G2 : Intubation rate: 12 (70.6%) Mortality 30 days: 5 (29.4%)	Not reported	Not reported
Garcia et al., 2022 ^(^ ^23^ ^)^ United States of America	G1 : Oxygen therapy G2 : HFNC G3 : NIV	N=11.826 G1 : 8.143 G2 : 2.859 G3 : 878	63.66 years old G1 : 64 (52-75) G2 : 64 (54-77) G3 : 63 (54-75)	G1 : 4.582/3.530 G2 : 1.683/1.159 G3 : 513/359	G1 : 29.2 (25.2-34.3) G2 : 28.9 (25.1-38.8) G3 : 29.5 (25.4-35.6)	G1 : CAD: 965 (11.9%), CHF: 617 (7.6%), COPD: 631 (7.7%), CKD: 1.236 (15.2%) and DM: 2.852 (35%) G2 : CAD: 339 (11.9%), CHF: 265 (9.3%), COPD: 232 (8.1%), CKD: 320 (11.2%) and DM: 869 (30.4%) G3 : CAD: 133 (15.1%), CHF: 128 (14.6%), COPD: 130 (14.8%), CKD: 138 (15.7%) and DM: 376 (42.8%)	Not reported	Not reported	G1 : Intubation rate: 1.830 (22.5%) Mortality: 1.302 (16%) G2 : Intubation rate: 838 (29.3%) Mortality: 811 (28.4%) G3 : Intubation rate: 290 (33%) Mortality: 294 (33.5%)	Not reported	G1 : SOFA score, median (IQR): 2 (1-6) G2 : SOFA score, median (IQR): 4 (2-6) G3 : SOFA score, median (IQR): 3 (1-6)
**Author, Year, Country**	**Intervention**	**Full Sample (N)/Sample per group**	**Age (years, yo) - total/ per group**	**Sex** **(M/F)**	**Body mass index (BMI)**	**Comorbidities**	**Medication**	**Intervention parameters**	**Primary outcomes**	**Secondary outcomes**	**Other results**
Wendel Garcia et al., 2022 ^(^ ^45^ ^)^ Spain	G1 : Oxygen therapy G2 : HFNC G3 : NIV	N=1.093 G1 : 553 G2 : 439 G3 : 101	63 years old G1 : 64 (55-71) G2 : 61 (52-69) G3 : 64 (57-70)	G1 : 374/179 G2 : 297/142 G3 : 68/33	G1 : 27.9 (25.6-31.2) G2 : 27.9 (26-31.2) G3 : 28.3 (25.8-32.7)	G1 : Cancer: 37 (7%), DM: 130 (24%), COPD: 47 (8%), Cardiovascular disease: 283 (51%) and Immunosuppression: 23 (4%) G2 : Cancer: 31 (7%), DM: 74 (17%), COPD: 22 (5%), Cardiovascular disease: 186 (42%) and Immunosuppression: 34 (8%) G3 : Cancer: 12 (12%), DM: 22 (22%), COPD: 10 (10%), Cardiovascular disease: 51 (50%) and Immunosuppression: 6 (6%)	Not reported	G1 : Oxygen mask greater than 10 L of oxygen/minute. G2 : Gas flow rate above 30L/minute and FiO_2_ of at least 0.5 G3 : NIV with FiO_2_ of at least 0.5 (regardless of interface, mode and type of ventilator used)	G1 : Intubation rate: 501 (91%) Mortality: 167 (30%) G2 : Intubation rate: 307 (70%) Mortality: 106 (24%) G3 : Intubation rate: 89 (88%) Mortality: 37 (36%)	Not reported	G1 : Length of hospital stay (days): 14 [7-26] G2 : Length of hospital stay (days): 13 [7-26] G3 : Length of hospital stay (days): 13 [8-24]
Alkouh et al., 2022 ^(^ ^46^ ^)^ Morocco	G1 : HFNC G2 : Oxygen therapy	N=233 G1 : 162 G2 : 71	65.49 years old G1 : 66.32±12.8 G2 : 64.66±14.97	G1 : 117/45 G2 : 49/22	G1 : 27.59 (± 12.8) G2 : 27.49 (± 4.93)	Not reported	Not reported	Not reported	G1 : Intubation rate: 80 (49.7%) Mortality: 79 (48.76%) G2 : Intubation rate: 33 (46.5%) Mortality: 34 (47.88%)	Not reported	Not reported
Domenico et al., 2020^(^ ^47^ ^)^ Italy	NIV/CPAP	N=310	Not reported	200/110	Not reported	SAH: 134 (43,5%), DM: 53 (17.7%), Heart disease: 50 (16.2%), Vascular disease: 34 (12.7%), COPD: 42 (13.5%, Immunosuppression or rheumatoid arthritis: 20 (6.4%)	Not reported	Not reported	Intubation rate: 177 (57%) Mortality: 90 (29%)	Pre therapy: PaO_2_/FiO_2_ 148mmHg Post therapy: PaO_2_/FiO_2_ : 248mmHg	Not reported
Burton et al., 2020 ^(^ ^48^ ^)^ United Kingdom	NIV	N=20	53.4±8.3 years old	11/9	Not reported	Lung disease: (35%), DM: (15%) and CHF (5%)	All: Antivirals: 20 (100%), Corticosteroids: 5 (20%)	Not reported	Intubation rate: 7 (35%) Mortality: 0 (0%)	.	Length of hospital stay: 11 days
Chandel et al., 2020^(^ ^49^ ^)^ United States of America	HFNC	N=272	56 years old	180/92	28.7 (25.2-33.4)	SAH: 116 (42.6%), DM: 101 (37.1), CKD: 20 (7.4), End-stage renal disease: 8 (2.9), CAD: 9 (3,3%), Hyperlipidemia: 74 (27.2%), Asthma: 13 (4.8%), COPD: 2 (0.7%), Active cancer: 7 (2.6%) and Systemic anticoagulation: 9 (3.3%)	Not reported	Not reported	Intubation rate: 109 (40%)	Not reported	Not reported
**Author, Year, Country**	**Intervention**	**Full Sample (N)/Sample per group**	**Age (years, yo) - total/ per group**	**Sex** **(M/F)**	**Body mass index (BMI)**	**Comorbidities**	**Medication**	**Intervention parameters**	**Primary outcomes**	**Secondary outcomes**	**Other results**
Vianello et al., 2020 ^(^ ^50^ ^)^ Italy	G1 : HFNC G2 : Early intubation	N=28	69 years old	21/7	Not reported	Patients with some comorbidity: (71.4%)	Not reported	G1 : Adjusted initially with 60L/min of flow and FiO_2_ 100% and then adjusted to keep SpO_2_>92%	G1 : Intubation rate: 11 (38%) Mortality: 4 (15%) G2 : Intubation rate: 100% Mortality: 21%	G1 : PaO_2_: 55.5 mmHg (39.9-61); PaCO_2_: 32.9 mmHg (27-39); PaO_2_/FiO_2 _: 76 (53-190). G2 : PaO_2_: 58.3 (36.2-7.1); PaCO_2_: 31.9 (28-45); PaO_2_/FiO_2_: 126 (52-296)	G1 : Time of days in the ICU: 11 days G2 : Time of days in the ICU: 20 days
Burnin et al., 2021^(^ ^51^ ^)^ United States of America	EG: HFNC CG: HFNC + Other Ventilation Strategies	N=3.125 EG: 504 CG: 2.621	62 years old EG: 64 (52-73) CG: 60 (44-74)	EG: 284/220 CG: 1.305/1.316	EG: 29.7 (25.6 - 35.3) CG: 28.6 (23.3 - 33.8)	EG: SAH: 16 (53.3%), CAD: 252 (50%), CHF: 161 (31%), CKD: 120 (23.8%), DM: 251 (49.8%), Asthma: 76 (15.1%), COPD: 177 (35.1%), Cancer: 54 (10.7%) and Liver disease: 76 (15.1%) CG: SAH: 273 (64.5%), CAD: 219 (51.8%), CHF: 108 (25.5%), CKD: 96 (22.7%), DM: 181 (42.8%), Asthma: 55 (13%) and COPD: 128 (30.3%)	EG: Hydroxychloroquine: 76 (15.1%), Azithromycin 243 (48.2%), Corticosteroids 318 (61.3%) and Remdesivir 257 (51%) CG: Hydroxychloroquine: 337 (12.9%), Azithromycin 829 (31.6%), Corticosteroids 802 (30.6%) and Remdesivir 539 (20.6%)	Not reported	EG: Mortality: 106 (25.2%) CG: Mortality: 79 (18.7%)	Not reported	Not reported
Roedl et al., 2020^(^ ^52^ ^)^ Germany	NIV or HFNC	N=57	63 years old	41/16	24.5 (23.1-27.1)	Not reported	All: Specific antiviral therapy: 2 (4%) and Glucocorticoid therapy: 5 (9%)	Not reported	Intubation rate: 46 (81%)	Not reported	Not reported
Pagano et al., 2020 ^(^ ^53^ ^)^ Italy	NIV	N=18	69 years old	13/5	Not reported	Not reported	Not reported	Not reported	Intubation rate: 8 (45%)	Therapy responders: PaO_2_/FiO_2_: 143±91 Non-responders to therapy: PaO_2_/FiO_2_: 167±72	Not reported
Daniel et al., 2021 ^(^ ^54^ ^)^ United States of America	G1 : Intubation as first option G2 : NIV G3 : NIV + Intubation	N=222 G1 : 91 G2 : 87 G3 : 44	69.5 (62-78) years old G1 : 67 (60-76) G2 : 67 (65-82) G3 : 69 (58-75)	129/93	29.39 (25.5-33) G1 : 30 (26-35) G2 : 28 (25-30) G3 : 31 (27-34)	G1 : DM: 49 (54%), SAH: 65 (71%), CAD: 10 (12%), Asthma: 8 (36%), COPD: 8 (35%) and Any Pulmonary disease: 19 (25%) G2 : DM: 52 (60%), SAH: 71 (83%), CAD: 20 (23%), Asthma: 11 (58%), COPD: 10 (53%) and Any Pulmonary disease: 24 (31%) G3 : DM: 35 (80%), SAH: 33 (77%), CAD: 7 (16%), Asthma: 3 (33%), COPD: 5 (42%) and Any Pulmonary disease: 9 (25%)	Not reported	Not reported	G1 : Mortality: 75 (82%) G2 : Mortality: 60 (69%) G3 : Mortality: 37 (84%)	Not reported	Not reported
**Author, Year, Country**	**Intervention**	**Full Sample (N)/Sample per group**	**Age (years, yo) - total/ per group**	**Sex** **(M/F)**	**Body mass index (BMI)**	**Comorbidities**	**Medication**	**Intervention parameters**	**Primary outcomes**	**Secondary outcomes**	**Other results**
Soares et al., 2020 ^(^ ^55^ ^)^ United States of America	CG: Intubation EG: HFNC, NIV and Prone position	N=469 CG: 254 EG: 215	69.5 years old CG: 68 EG: 71	CG: 130/124 EG: 99/116	CG: 30.8 EG: 30.1	CG: DM: (11%), Chronic Pneumopathy: (33.9%) and CHF: (32.7%) EG: DM: (7.9%), Chronic Pneumopathy: (35.8%) and CHF: (29.3%)	Not reported	Not reported	CG: Intubation rate: 74 (29%) Mortality: 56 (22%) EG: Intubation rate: 30 (14%) Mortality: 47 (22%)	Not reported	Not reported
Duca et al., 2020 ^(^ ^56^ ^)^ Italy	CPAP	N=71	70 years old	61/10	Not reported	SAH: (58%), DM: (23%), CHF: (8.5%) and COPD: (5.6%)	Not reported	Not reported	Intubation rate: 26 (37%) Mortality: 54 (76%)	PaO_2_: 85mmHg (69-123)* PaCO_2_: 34mmHg (31-37)* PaO_2_/FiO_2_: 131 (97-190)* (average and IQR)*	Not reported
López-Padilla et al., 2021^(^ ^57^ ^)^ Spain	NIV or HFNC	N=82	58.8 years old	55/27	Not reported	SAH: (41.5%), DM: (24.4%), CHF: (15%), COPD: (3.7%) and Ischemic Heart Disease: (8.5%)	All: Hydroxychloroquine: 80 (98%), Ritonavir: 78 (95%), Azithromycin: 21 (24.7%), Corticosteroids: 67 (78.8%) and Tocilizumab: 20 (23.5%)	Not reported	Intubation rate: 17 (21%) Mortality: 10 (12%)	SpO_2_/FiO_2_:121.7 (60.01)	Time of Days of Symptoms (median days) 11 (8-15)
Case Series											
Giron et al., 2020 ^(^ ^58^ ^)^ United States of America	HFNC + Non-rebreathing mask	N=4	60.25±years old	2/2	Not reported	SAH: (50%) and DM: (50%)	All: Hydroxychloroquine: 4 (100%), Azithromycin: 4 (100%)	HFNC: 10-15L/min Non-rebreathing mask: 15L/min and prone position	Intubation rate: 2 (50%) Mortality rate: 0 (30 days)	Case one: PaO_2_: 70mmHg; PaCO_2_: 32mmHg; PaO_2_/FiO_2_: 77.78 Severe ARDS classification by the Berlin criteria. Case two: PaO_2_: 64mmHg; PaCO_2_: 34mmHg; PaO_2_/FiO_2_: 71 Severe ARDS classification by the Berlin criteria. Case three: PaO_2_: 111mmHg; PaCO_2_: 36mmHg PaO_2_/FiO_2_: 123. Moderate ARDS classification by the Berlin criteria Case four: PaO_2_: 62mmHg; PaCo_2_: 34mmHg; PaO_2_/FiO_2_: 68.89. Severe ARDS classification according to the Berlin criteria	Length of hospital stay: 21. 75 days

M/F: Male / Female; BMI: Body Mass Index; G1: Group one; G2: Group two; G3: Group three; CG: Control group or comparison group; EG: Experimental group; HFNC: High flow nasal cannula; CPAP: Continuous Positive Airway Pressure; NIV: Non-invasive mechanical ventilation; BIPAP: Bilevel positive airway pressure; SAH: systemic arterial hypertension; DM: Diabetes mellitus; CKD: Chronic kidney failure; COPD: Chronic obstructive pulmonary disease; CHF: Congestive heart failure; AMI: Acute myocardial infarction; CAD: Coronary artery disease; CD: Heart disease; IRpC: Chronic respiratory failure; HF: Heart failure; ARDS: Acute Respiratory Distress Syndrome; ICU: Intensive care unit; PaCO_2_: Blood pressure of carbon dioxide; PaO_2_/FiO_2_: Relationship between arterial oxygen pressure and fraction of inspired oxygen; SpO_2_: Peripheral oxygen saturation; PaO_2:_ Blood pressure of oxygen; FiO_2_: Fraction of inspired oxygen; PEEP: Positive end-expiratory pressure; PS: Pressure support; SpO_2_/FiO_2_: Relationship between peripheral oxygen saturation and fraction of inspired oxygen; SOFA: Sequential assessment of organ failure; APACHE: Acute physiology and chronic health evaluation; IQR: interquartile range.

Most included study designs were observational (85.4%) and primarily employed HFNC therapy (85.4%). Sample sizes ranged from 4 to 11,826 participants, with or without control and intervention groups, totaling 23,901 participants (60.3% men, mean age: 62.9 years).

The most investigated therapies were HFNC *versus* NIV (26.9%), followed by HFNC *versus* conventional oxygen therapy (24.5%), and HFNC combined with NIV (21.9%), For 32% and 22% of the studies, progression to intubation and a high mortality rate were reported, respectively. Participants who received HFNC had a mean PaO_2_/FiO_2_ ratio of 166.07, whereas those who received NIV had a mean PaO_2_/FiO_2_ ratio of 147.82.

The authors of only 14 studies (34.14%)^( 16 - 18 , 28 , 32 , 34 , 35 , 42 , 44 , 48 , 51 , 52 , 57 , 58 )^ reported the use of pharmacological agents as adjuvants in disease treatment, including hydroxychloroquine and steroids (35.71%) and corticosteroids and azithromycin (28.25%). However, no subgroup analysis was conducted to evaluate the effects of concomitant medication use with HFNC.

### Meta-analysis results

A meta-analysis ( Table 1 ) was conducted on five RCTs and five cohort studies. HFNC studies revealed intubation rates from 29% to 51% and mortality rates ranging from 0% to 50%. Non-invasive ventilation studies revealed an intubation rate of 30% to 79.6% and a mortality rate ranging from 22% to 61% ( Table 2 ).

**Table 1:** Descriptive characteristics of the studies included in the meta-analysis

Authors, year, country	Intervention	Total sample (N)/sample per group	Age (years) – median total/per group – median (range) or mean±SD	Sex by group	Body mass index, kg/m^2^, median (range) or mean±SD	Comorbidities	Medication
**Randomized clinical trials**							
Grieco et al., 2021 ^(^ ^11^ ^)^ Italy	G1: NIV helmet interface G2: HFNC	n=109 G1 : 54 G2 : 55	64.5 years old G1: 66 (57-72) G2: 63 (55-69)	G1: Male: 42 (77%) / Female: 12 (23%) G2: Male: 46 (84%)/ Female: 9 (16%)	G1: 27 (26-30) G2: 28 (26-31)	G1: SAH: 24 (44%), DM: 13 (24%), smoking: 5 (9%), immunosuppression: 3 (6%), cancer: 2 (4%), and HIV: 1 (2%) G2 : SAH: 33 (60%), DM: 10 (18%), smoking: 11 (20%), immunosuppression: 5 (9%), and HIV: 1 (2%)	Not reported
Teng et al., 2021 ^(^ ^12^ ^)^ China	G1: HFNC G2: Conventional oxygen therapy	n=22 G1: 12 G2: 10	55 years old G1: 56.6±3.0 G2 : 53.5±5.5	G1 : Male: 4 (33.4%) / Female: 8 66.66%) G2 : Male: 7 (70%) / Female: 3 (30%)	G1 : 31.9 G2 : 30.8	G1: CKD: 12 (42.9%), COPD: 7 (25%), DM: 12 (53.6%), SAH: 20 (71.4%)and AMI: 6 (21.4%) G2: CKD: 43 (45.3%), COPD: 27 (28.4%), DM: 45 (47.4%), SAH: 83 (87.4%) and AMI: 22 (23.2%)	Not reported
Nair et al., 2021 ^(^ ^13^ ^)^ India	G1 : HFNC G2 : NIV	n=109 G1 : 55 G2: 54	57.25 years old G1 : 57 (48-65) G2: 57.5 (47-64)	G1 : Male: 44 (80%) / Female: 11 (20%) G2 : Male: 35 (64.8%) / Female: 19 (35.2%)	Not reported	G1: DM: 17 (30.90%), SAH: 17 (30.90%), CAD: 10 (18.18%), CKD: 4 (7.27%), and CLD: 1 (1.85%) G 2: DM: 16 (29.62%), SAH: 20 (37.03%), CAD: 7 (12.96%), CKD: 12 (22.22%), and CLD: 1 (1.85%)	Not reported
Ospina-Tascón et al., 2021 ^(^ ^14^ ^)^ Colombia	G1: HFNC G2 : Oxygen therapy	n=199 G1: 99 G2: 100	59.5 years old G1: 60 (50-69) G2: 59 (49-67)	G1: Male: 71 (72%) / Female: 28 (28%) G2: Male: 63 (63%)/ Female: 37 (37%)	Not reported	G1: SAH: 35 (35%), DM: 18 (18%), COPD: 3 (3%), CD: 3 (3%) and Cancer: 1 (1%) G2: SAH: 44 (44%), DM: 20 (20%), COPD: 1 (1%), CKD: 1 (1%), and CD: 4 (4%)	Not reported
Perkins et al., 2022 ^(^ ^15^ ^)^ United Kingdom	G1 : NIV CPAP G2: HFNC G3: Oxygen therapy	n=1,273 G1 : 380 G2 : 418 G3: 475	57.3 years old G1: 56.7 (12.5) G2: 57.6 (13.0) G3 : 57.6 (12.7)	G1: Male: 260 (31.6%) / Female: 120 (68.4%) G2: Male: 272 (65.1%) / Female: 146 (34.9%) G3: Male: 312 (65.7%) / Female: 163 (34.3%)	Not reported	G1: SAH: 131 (34.5%), DM: 86 (22.6%), and other: 148 (38.9) G2: SAH: 164 (39.2%), DM: 98 (23.4%), and other: 141 (33.7) G3 : SAH: 153 (32.2%), DM: 91 (19.2%), and other: 188 (39.6)	Not reported
**Cohort**							
Franco et al., 2020 ^(^ ^16^ ^)^ Italy	G1: HFNC G2: CPAP G3: NIV	n=670 G1: 163 G2: 330 G3: 177	68.26 years old G1: 65.7 G2: 70.3 G3: 66.8	G1: Male: 114 (70%) / Female: 49 (30%) G2: Male: 225 (68%) / Female: 105 (32%) G3: Male: 127 (72%) / Female: 50 (28%)	Not reported	G1: SAH 74 (45.4%), DM: 32 (19.6%), obesity: 33 (24.3%), COPD: 9 (5.5%), chronic cardiovascular disease: 29 (187.8%) and Cancer: 17 (10.7%) G2 : SAH 153 (46.4%), DM: 60 (18.2%), obesity: 31 (9.8%), COPD: 12 (3.6%), chronic cardiovascular disease: 54 (16.4%), and cancer: 2 (0.6%) G3: SAH: 84 (47.4%), DM: 33 (18.7%), obesity: 44 (24.8%), COPD: 25 (14.1%), chronic cardiovascular disease: 22 (14.4%), and cancer: 8 (4.5%)	G1: Hydroxychloroquine 128 (78.5%), Lopinavir or ritonavir: 58 (35.6%), darunavir or cobicistat: 37 (22.7%), remdesivir: 1 (0.6%), tocilizumab: 47 (28.8%), methylprednisolone: 105 (64.4%), prophylaxis-LMWH: 63 (38.6%), and therapeutic-LMWH: 69 (42.3%) G2: Hydroxychloroquine: 108 (32.7%), lopinavir or ritonavir: 10 (3%), darunavir or cobicistat: 9 (2.7%), remdesivir: 1 (0.3%) tocilizumab: 65 (19.7%), methylprednisolone: 113 (34.2%), prophylaxis-LMWH: 20 (6%), and therapeutic-LMWH: 48 (14.5%) G3: Hydroxychloroquine: 27 (15.2%), lopinavir or ritonavir: 6 (3.4%), darunavir or cobicistat: 1 (0.6%), remdesivir: 0 (0%), tocilizumab: 49 (27.7%), methylprednisolone: 57 (32.2%), prophylaxis-LMWH: 7 (4%) and therapeutic-LMWH: 13 (7.3%)
**Authors, year, country**	**Intervention**	**Total sample (N)/sample per group**	**Age (years) – median total/per group – median (range) or mean±SD**	**Sex by group**	**Body mass index, kg/m^2^, median (range) or mean±SD**	**Comorbidities**	**Medication**
Hansen et al., 2021 ^(^ ^17^ ^)^ USA	G1: HFNC G2: Conventional oxygen therapy	n=92 G1: 30 G2: 61	60 years old G1: 68.6±12.5 G2: 68.13±11.9	G1: Male: 21 (70%) / Female: 9 (30%) G2: Male: 37 (59.7%) / Female: 25 (40.3%)	G1: 32.2±8.1 G2: 31.4±9.8	G1: CKD: 6 (20%), SAH: 16 (53.3%), DM: 9 (30%), cancer: 4 (13.3%), COPD: 6 (20%), asthma: 1 (3.3%), and insanity: 2 (6.7%) G2: CKD: 14 (22.6%), SAH: 45 (72.6%), DM: 27 (43.6%), Cancer: 10 (16.1%), COPD: 6 (9.7%), asthma: 5 (8.1%), and insanity: 11 (17.7%)	G1: hydroxychloroquine: 17 (56.7%), tocilizumab: 7 (23.3%), convalescent plasma: 1 (3.3%) statin: 25 (83.3%), azithromycin: 7 (23.3%), and steroids or corticosteroids: 24 (80%) G2: Not reported
Wendel Garcia et al., 2021 ^(^ ^18^ ^) ^Switzerland	G1: Oxygen therapy G2: HFNC G3: NIV	n=259 G1: 85 G2: 87 G3: 87	64 years old G1: 63 (53-72) G2: 63 (55-74) G3: 66 (55-76)	G1: Male: 63 (75%) / Female: 22 (25%) G2: Male: 65 (75%) / Female: 22 (25%) G3: Male: 62 (71%) / Female: 25 (29%)	G1 : 28 (26-32) G2: 27 (25-32) G3 : 26 (24-29)	G1: SAH: 42 (49%), CD: 11 (12%), DM: 23 (27%), COPD: 14 (16%), and others: 7 (8%) G2: SAH: 34 (39%), CD: 7 (8%), DM: 26 (29%), COPD: 10 (11%) and others: 13 (14%) G3: SAH: 36 (41%), CD: 10 (11%), DM: 17 (19%), COPD: 7 (8%), and others: 7 (8%)	G1: Not reported G2: Steroids or corticosteroids: 13 (29%) G3: Steroids or corticosteroids: 12 (28%)
Gough et al., 2021 ^(^ ^19^ ^)^ Ireland	G1 : CPAP G2: HFNC G3: Oxygen therapy	n=164 G1 : 85 G2 : 32 G3: 47	66.33 years old G1 : 61 (51-73) G2 : 73 (56-93) G3: 62 (48-75)	G1: Male: 37 (43.4%) / Female: 48 (56.6%) G2: Male: 17 (51.6%) / Female 15: (48.%) G3: Male: 29 (60.9%) / Female: 18 (39.1%)	G1 : 29.7 (27-34) G2 : 28.7 (25-35) G3 : 31 (23-38)	G1: respiratory disease: 23 (31,5%), heart disease: 21 (28,8%), DM: 10 (13,7%), and SAH: 38 (52,1%) G2 : respiratory disease: 8 (36.4%), heart disease: 5 (36,4), DM: 5 (22,7%), and SAH: 12 (54,5%) G3: respiratory disease: 16 (41%), heart disease: 14 (35,9%), DM: 12 (30.8) and SAH: 20 (51,3%)	Not reported
COVID-ICU group, for the REVA network, COVID-ICU investigators. 2021 ^(^ ^20^ ^)^ France/Belgium/Switzerland	G1: Oxygen therapy G2: HFNC G3: NIV	n=1.491 G1: 766 G2: 567 G3: 158	63.33 years old G1: 61 (53-70) G2 : 64 (55-72) G3: 65 (56-72)	G1 : Male: 554 (72%) / Female: 212 (28%) G2: Male: 427 (75%) / Female: 140 (25%) G3: Male: 113 (71%) / Female: 45 (29%)	G1: 28 (25-32) G2: 28 (25-31) G3: 29 (26-33)	G1: SAH: 331 (43%), DM: 206 (27%), Smokers: 35 (5%) and immunodeficiency: 42 (6%) G2: SAH: 263 (47%), DM: 145 (26%), smokers: 19 (4%) and immunodeficiency: 48 (9%) G3: SAH: 93 (59%), DM: 58 (37%), smokers: 13 (8%) and immunodeficiency: 12 (8%)	Not reported

G1: Group one; G2: Group two; G3: Group three; NIV: Non-invasive mechanical ventilation; HFNC: High-flow nasal cannula; CPAP: Continuous positive airway pressure; SD, Standard deviation; SAH: Systemic arterial hypertension; DM: Diabetes mellitus; HIV: Human immunodeficiency virus; CKD: Chronic kidney failure; COPD: Chronic obstructive pulmonary disease; AMI: Acute myocardial infarction; CAD: Coronary artery disease; CLD: Chronic liver disease; CD: Cardiovascular disease; LMWH, Low-molecular-weight heparin.

**Table 2:** Primary and secondary outcomes of the studies included in the meta-analysis

Authors, year	Intervention	Intervention parameters	Primary outcomes	Secondary outcomes	Other results
Grieco et al., 2021 ^(^ ^11^ ^)^	G1 : NIV interface helmet G2 : HFNC	G1 : NIV with compressed gas-based ventilator with bi-tube circuit. Pressure support mode adjusted between 10 and 12cmH_2_O to ensure a peak inspiratory flow of 100L/min; PEEP between 10 and 12cmH_2_O; and FiO_2_ to obtain an SpO_2_ between 92% and 98% G2 : Flow initially set to 60L/min at 37°C or 34°C, reduced in case of intolerance; and FiO_2_ to obtain SpO_2_ between 92% and 98%.	G1 : Intubation rate: 16 (30%) Mortality rate: 8 (15%) over 28 days / 13 (24%) over 60 days ICU mortality: 11 (20%) Hospital mortality: 13 (24%) G2 : Intubation rate: 28 (51%) Mortality rate: 10 (18%) over 28 days / 12 (22%) over 60 days ICU mortality: 14 (25%) Hospital mortality: 14 (25%)	G1 : Hypoxemia: 15 (28%) Dyspnea: 9 (17%) G2 : Hypoxemia: 27 (49%) Dyspnea: 25 (45%)	G1 : Signs of fatigue of the respiratory muscles: 13 (24%) Median (Q1-Q3) length of stay: ICU: 9 (4-17) days Hospital: 21 (14-30) days G2 : Signs of fatigue of the respiratory muscles: 24 (44%) Median (IQR) length of stay: ICU: 10 (5-23) days Hospital: 21 (13-44) days
Teng et al., 2021 ^(^ ^12^ ^)^	G1 : HFNC G2 : Conventional oxygen therapy	G1 : Initial parameters of the HFNC: flow: 50L/min, FiO_2_: 0.5, and temperature: 37°C, titrated by SpO_2_ level (target >93%), blood gas analysis, and tolerance. Duration of treatment >72 h. G2 : Nasal catheter or common mask (venturi and reservoir mask): initial flow fixed at 5L/min targeting SpO_2_ >93%. Duration of treatment >72 h.	All patients in this study were cured and discharged; 0% mortality in both groups.	G1 : Mean±SD PaO_2_/FiO_2_: 224.25±12.60 (at the start of treatment) 269.00±0.901 (after 6 h of treatment) 269.50±6.61 (24 h) 320.92±4.79 (72 h) G2 : Mean±SD PaO_2_/FiO_2_: 216.70±4.62 (0 h) 238.50±7.32 (6 h) 261.60±8.16 (24 h) 286.40±7.29 (72 h)	G1 : Mean±SD length of stay in the ICU: 4.00±0.74 days Total length of stay: 14.67±1.97 days G2 : Length of ICU stay: 4.90±1.0 days Total length of stay: 16.60±2.54 days
Nair et al., 2021 ^(^ ^13^ ^)^	G1 : HFNC G2 : NIV	G1 : HFNC with initial gas flow set at 50L/min and FiO_2_ at 1.0. Flow and FiO_2_ were subsequently adjusted to 30-60L/min and 0.5-1.0, respectively, to maintain an SpO_2_ ≥94%. G2 : NIV applied with a mask/helmet connected to the ventilator in pressure support mode of 10-20cmH_2_O, to obtain an exhaled tidal volume of 7-10 mL per kg predicted body weight, a PEEP of 5-10cmH_2_O, and 0.5-1.0 FiO_2_ titrated to target an SpO_2_ >94%.	G1 : Intubation rate in 48 h: 11 (20%) / by day 7: 15 (27.3%) Mortality: 16 (29.1%) G2 : Intubation rate in 48 h: 18 (33.3%) / by day 7: 25 (46%) Mortality: 25 (46%)	G1 : Median (range) PaO_2_: 61 (52.5-83.6) mmHg Median (range) PaCO_2_: 34 (26.3-38.5) mmHg Median (range) PaO_2_/FiO_2_: 105 (92-139.3) G2 : Median (range) PaO_2_: 64.5 (52.9-85.6) mmHg Median (range) PaCO_2_: 32 (26-43.2) mmHg Median (range) PaO_2_/FiO_2_: 111.1 (89.8-145)	G1 : Median (range) hospital stay: 9 (7-13) days G2 : Hospital stay: 9 (6-12) days
Ospina-Tascón et al., 2021 ^(^ ^14^ ^)^	G1 : HFNC G2 : Oxygen therapy	G1 : Initial HFNC: flow rate of 60 L/min with an FiO_2_ of 1.0 to maintain an SpO_2_ ≥92%. Flow was decreased in patients who reported discomfort. G2 : Oxygen was continuously applied through any low-flow device or combination (nasal prongs, mask with or without oxygen reservoir, or venturi systems) to maintain an SpO_2_ ≥92%	G1 : Intubation rate, 28 days: 34 (34%) Mortality by day 14: 6 (6%) / day 28: 8 (8%) G2 : Intubation rate, 28 days: 51 (51%) Mortality by day 14: 6 (6%) / day 28: 16 (16%)	G1 : Median (range) PaO_2_: 78 (66-97) mmHg Median (range) PaCO_2_: 32 (30-35) mmHg Median (range) PaO_2_/FiO_2_: 104 (85-132) G2 : Median (range) PaO_2_: 73 (63-92) mmHg Median (range) PaCO_2_: 32 (30-36) mmHg Median (range) PaO_2_/FiO_2_: 105 (85-141)	Clinical recovery in 28 days G1 : 77 (77.8) G2 : 71 (71.0) Time to clinical recovery, median (range) G1 : 11 (9-14) days G2 : 14 (11-19) days
Perkins et al., 2022 ^(^ ^15^ ^)^	G1 : NIV CPAP G2 : HFNC G3: Oxygen therapy	G1 : PEEP: 8.8cmH_2_O G2 : Flow rate: 52.4L/min G3: Not reported	G1 : Intubation rate: 126 (33.4%) Mortality: 63 (16.7%) G2 : Intubation rate: 170 (41%) Mortality: 78 (18.8%) G3: HFNC: Intubation rate: 153 (41.6%) Mortality: 74 (20%) CPAP: Intubation rate: 147 (41.3%) Mortality: 69 (19.2%)	G1 : Median (range) PaO_2_: 67.5 (60-77.3) mmHg Median (range) PaCO_2_: 33 (30-36.8) mmHg Median (range) PaO_2_/FiO_2_: 112.5 (80-161.3) G2 : Median (range) PaO_2_: 66 (59.3-74.3) mmHg Median (range) PaCO_2_: 33 (30-36) mmHg Median (range) PaO_2_/FiO_2_: 115 (80.9-168.4) G3: Median (range) PaO_2_: 66.8 (58.5-80.3) mmHg Median (range) PaCO_2_: 33.8 (30.8-36.8) mmHg Median (range) PaO_2_/FiO_2_: 113.8 (84.8-150.9)	Not reported
**Authors, year**	**Intervention**	**Intervention parameters**	**Primary outcomes**	**Secondary outcomes**	**Other results**
Franco et al., 2020 ^(^ ^16^ ^)^	G1 : HFNC G2 : CPAP G3: NIV	G1 : Mean flow rate: 50.5L/min (SD: 8) G2 : Mean PEEP: 10.2cmH_2_O (SD: 1.6) via helmet or face mask G3: Mean PEEP: 9.5cmH_2_O (SD: 2.2) via helmet or face mask; mean pressure support: 17.3cmH_2_O (SD: 3.0)	G1 : Intubation rate: 47 (29%) Mortality: 26 (16%) G2 : Intubation rate: 82 (25%) Mortality: 100 (30.3%) G3: Intubation rate: 49 (28%) Mortality: 54 (30.5%)	G1 : Mean±SD PaO_2_/FiO_2_: 166±65 G2 : Mean±SD PaO_2_/FiO_2_: 151±90 G3: Mean±SD PaO_2_/FiO_2_: 138±66	Not reported
Hansen et al., 2021 ^(^ ^17^ ^)^	G1 : HFNC G2 : Conventional oxygen therapy	G1 : Flow rate: 20-60L/min, with FiO_2_ ranging from 0.21 to 1.0, targeting an SpO_2_ ≥92% G2 : Received oxygen through a non-rebreathing mask or a reservoir nasal cannula, with an oxygen flow of 10-15L/min	G1 : Mortality: 9 (30%) G2 : Mortality: 33 (53.2%)	G1 : PaO_2_/FiO_2_ initial: mean±SD: 152±62 G2 : PaO_2_/FiO_2_ initial: mean±SD: 153±67	G1 : Mean±SD SOFA score: 6.6±2.2 G2 : Mean±SD SOFA score: 7.7±3.0
Wendel Garcia et al., 2021 ^(^ ^18^ ^)^	G1 : Oxygen therapy G2 : HFNC G3: NIV	G1 : Not reported G2 : Flow rate >30L/min; mean FiO_2_: 44%-80% G3: Not reported	G1 : Mortality: 15 (18%) G2 : Mortality: 17 (20%) G3: Mortality: 32 (37%)	G1 : Median (range) PaO_2_/FiO_2_: 117 (105-160) G2 : Median (range) PaO_2_/FiO_2_: 126 (79-169) G3: Median (range) PaO_2_/FiO_2_: 135 (97-168)	G1 : Mean±SD ICU stay: 9±3.17 days G2 : ICU stay: 13±6.24 days G3: ICU stay: 17±8.26 days
Gough et al., 2021 ^(^ ^19^ ^)^	G1 : CPAP G2 : HFNC G3 : Oxygen therapy	Not reported	G1 : Mortality: 49 (57.7%) G2 : Mortality: 16 (50%) G3: Mortality: 28 (60%)	G1 : Median (range) PaO_2_: 60 (52.3-70.7) mmHg Median (range) PaCO_2_ :33.4 (30.4-37.8) mmHg Median (range) PaO_2_/FiO_2_: 191.3 (108-242.3) G2 : Median (range) PaO_2_: 63.8 (55.9-80.9) mmHg Median (range) PaCO_2_: 34.4 (29.5-44.6) mmHg Median (range) PaO_2_/FiO_2_: 183 (82.5-275.3) G3: Median (range) PaO_2_: 66.8 (56.6-77.3) mmHg Median (range) PaCO_2_: 32.8 (29.3-37.1) mmHg Median (range) PaO_2_/FiO_2_: 286.5 (225-351.8)	Not reported
COVID-ICU group, for the REVA network, COVID-ICU investigators. 2021 ^(^ ^20^ ^)^	G1 : Oxygen therapy G2 : HFNC G3: NIV	Not reported	G1 : Intubation rate: 359 (47%) Hospital mortality: 127 (17%) ICU mortality: 108 (14%) G2 : Intubation rate: 242 (43%) Hospital mortality: 118 (22%) ICU mortality: 109 (19%) G3: Intubation rate: 77 (49%) Hospital mortality: 62 (40%) ICU mortality: 52 (33%)	Not reported	G1 : SOFA score range: 2-4 G2 : SOFA score range: 2-4 G3: SOFA score range: 2-5

G1: Group one; G2: Group two; G3: Group three; NIV: Non-invasive mechanical ventilation; HFNC: High-flow nasal cannula; CPAP: Continuous positive airway pressure; PEEP: Positive end-expiratory pressure; FiO_2_: Fraction of inspired oxygen; SpO_2_: Peripheral oxygen saturation; ICU: Intensive care unit; Q: quartile; PaO_2_/FiO_2_: Relationship between arterial oxygen pressure and fraction of inspired oxygen; PaO_2:_ Arterial blood pressure of oxygen; PaCO_2_: Arterial blood pressure of carbon dioxide; SD: Standard deviation; SOFA: Sequential assessment of organ failure.

**Figure 2:**
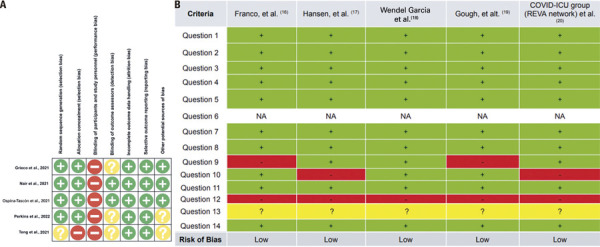
Risk analysis of studies included in the meta-analysis. A) Randomized controlled trials assessed via the Cochrane Risk of Bias Tool. B) Cohort studies assessed via the Quality Assessment Tool for Observational Cohort and Cross-Sectional Studies of the National Institutes of Health (NIH, USA), classified by cell background in shades of green: yes, red: no, and yellow: cannot be determined NA: not applicable.

Risk of bias assessment for the five RCTs^( 11 - 15 )^indicated a low risk of attrition and reporting bias ( Figure 2 ). The quality assessment of the five cohort studies in the meta-analysis was conducted using the NIH Quality Assessment Tool, which indicated a low risk of bias ( Figure 2 ).

### Intubation rate

**Figure 3:**
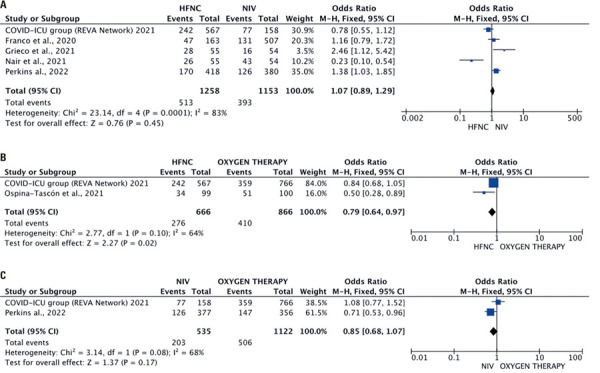
Forest plots of intubation rates. A) Comparison of high-flow nasal cannula (HFNC) and non-invasive ventilation (NIV); B) Comparison of HFNC and conventional oxygen therapy; C) Comparison of NIV versus conventional oxygen therapy. Data expressed as Mantel-Haenszel (M-H) odds ratios (ORs) with fixed and random effects, along with the 95% confidence intervals (95%CIs)

The intubation rate for HFNC *versus* NIV was analyzed in five studies^( 11 , 13 , 15 , 16 , 20 )^involving 2,411 participants (HFNC: 1,258; NIV: 1,153). The pooled OR was 1.07 (95%CI=0.89-1.29), with high heterogeneity (I^2^= 83%; Figure 3A ) and no difference between the groups (p=0.45).

The intubation rate was reported in two studies (1,532 participant) for HFNC (666 participants) *versus* conventional oxygen therapy (866 participants).^( 14 , 20 )^ The pooled OR of 0.79 (95%CI=0.64-0.97; Figure 3B ) indicates a protective effect of HFNC against intubation compared with conventional oxygen therapy (p=0.02).

Two studies^( 15 , 20 )^ of NIV (535 participants) *versus* conventional oxygen therapy (1,122 participants) revealed no difference in the intubation rate (M-H OR: 0.85, 95%CI=0.68-1.07, p=0.17; Figure 3C ).

### Mortality rate

**Figure 4:**
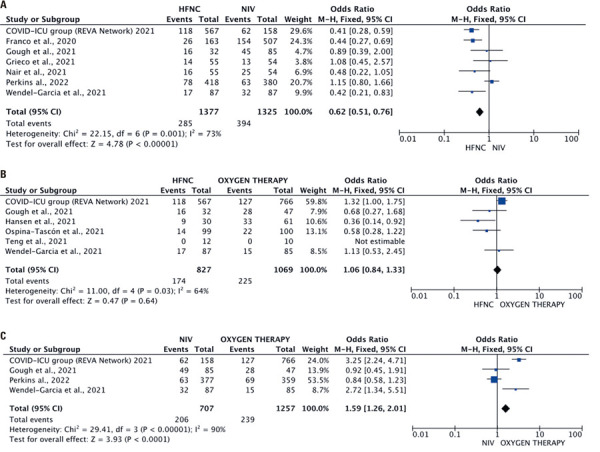
Forest plots of hospital mortality rates. A) Comparison of high-flow nasal cannula (HFNC) and non-invasive ventilation (NIV); B) Comparison of HFNC and conventional oxygen therapy; C) Comparison of NIV and conventional oxygen therapy. Data expressed as Mantel-Haenszel (M-H) odds ratios (ORs) with fixed and random effects, along with 95% confidence intervals (CIs)

The mortality rate was analyzed in seven studies^( 11 , 13 , 15 , 16 , 18 - 20 )^ involving 2,702 participants (HFNC: 1,377; NIV: 1,325). The pooled OR was 0.62 (95%CI=0.51-0.76), with moderate heterogeneity (I^2^=73%; Figure 4A ), favoring HFNC (p<0.0001).

No significant differences were observed in mortality rates across six studies^( 12 , 14 , 17 - 20 )^including 1,896 participants (HFNC: 827; conventional oxygen therapy: 1,069). The pooled OR was 1.06 (95%CI=0.84-1.33, p=0.64), with moderate heterogeneity (I^2^=64%; Figure 4B ).

The mortality rate was reported in four studies^( 15 , 18 - 20 )^ for NIV (707 participants) *versus* conventional oxygen therapy (1,257 participants), with high heterogeneity (I^2^=90%). The analysis favored conventional oxygen therapy (OR=1.59, 95%CI=1.26-2.01, p<0.0001; Figure 4C ).

## DISCUSSION

The meta-analysis yielded three key findings. (1) No significant difference was observed between HFNC and NIV in terms of the reduction in the intubation rate. However, HFNC was superior to conventional oxygen therapy, whereas NIV yielded a similar response to conventional oxygen therapy. (2) HFNC was associated with a lower mortality rate than NIV. (3) Finally, the mortality rate was similar between HFNC and conventional oxygen therapy, whereas conventional oxygen therapy was associated with a lower mortality rate than NIV. However, these results were influenced by the heterogeneity of studies and clinical characteristics of the participants.

### Intubation rate

The meta-analysis of the intubation rate included five studies,^( 11 , 13 , 15 , 16 , 20 )^ with a total of 2,411 participants (HFNC: 1,258; NIV: 1,153; OR=1.07, 95%CI=0.89-1.29), revealing no significant difference between HFNC and NIV. A previous systematic review^( 6 )^ revealed an estimated OR of 1.21 (95%CI=0.45-3.29, p=0.71), also without a significant difference. This may indicate equivalence between the therapies for this outcome. The numeric risk values differ owing to the inclusion of different studies, with a total of 906 events (n=2,411) compared with 184 events (n=380). This discrepancy can also be attributed to differences in study selection, as we included only RCTs and cohort studies. In contrast, He et al.^( 6 )^ included only one RCT; and the rest were retrospective studies.

A possible explanation for these findings is that both HFNC and NIV provide effective respiratory support, thereby reducing the need for IMV. Both strategies may alleviate respiratory distress, improve oxygenation, and prevent further clinical deterioration. However, HFNC was superior to conventional oxygen therapy in terms of oxygenation and respiratory support. It may reduce the severity of respiratory failure and the subsequent need for intubation.

Franco et al.^( 16 )^ demonstrated the feasibility and clinical impact of non-invasive respiratory support in patients with COVID-19. Their findings indicate that HFNC and NIV yield comparable intubation rates, suggesting that both strategies effectively provide respiratory support and prevent the need for IMV. However, their meta-analysis highlights the superiority of HFNC over conventional oxygen therapy in the prevention of intubation, a finding supported by Ospina-Tascón et al.,^( 14 )^ who reported a reduced need for IMV with HFNC compared with conventional oxygen therapy. A possible explanation is that HFNC, by delivering a high flow of oxygen and increasing the end-expiratory lung volume, improves oxygenation and reduces the work required to breathe more effectively than conventional oxygen therapy. This may lead to improved outcomes by preventing the progression of respiratory failure and reducing the subsequent need for intubation.

Supporting these findings, He et al^( 6 )^ evaluated three studies involving 101 patients in their meta-analysis, demonstrating an improvement in the PaO_2_/FiO_2_ ratio at 24 h in the HFNC group (p<0.00001). Conversely, Peng et al.^( 21 )^ found no differences in the PaO_2_/FiO_2_ ratio between the HFNC and other non-invasive respiratory strategies (p=0.07). Given the variability in study quality and methodologies, uncertainties remain regarding the optimal application of non-invasive respiratory strategies.

Notably, substantial heterogeneity exists in the sample sizes and distribution of participants across studies. Perkins et al.^( 15 )^included a larger cohort than the other researchers,^( 11 , 13 , 16 , 20 )^ potentially leading to an overestimation of the favorable effects of HFNC and NIV on the intubation rate.

Compared with conventional oxygen therapy, HFNC demonstrated superior efficacy in patients with severe symptoms, likely owing to its ability to generate positive airway pressure.^( 22 )^ The physiological effects associated with such positive pressure may explain the observed decrease in intubation rates among patients critically ill with COVID-19 by improving respiratory function and alleviating symptoms. Two studies^( 14 , 20 )^included in our meta-analysis favored HFNC over conventional oxygen therapy (OR=0.79, 95%CI=0.64-0.97). Sayan et al.^( 22 )^also reported that HFNC use in cases of respiratory failure significantly reduced intubation rates.

### Mortality rate

Our meta-analysis of mortality rates across seven studies^( 11 , 14 , 15 , 16 , 18 - 20 )^ with a total of 2,702 participants (HFNC: 1,377; NIV: 1,325) demonstrated a significantly lower risk associated with HFNC than that with NIV. This finding aligns with that of several previous studies. Garcia et al.^( 23 )^highlighted variability in hospital practices regarding the use of HFNC and NIV for acute respiratory failure secondary to COVID-19, suggesting a potential mortality benefit with HFNC. Similarly, regarding the HENIVOT clinical trial, Grieco et al.^( 11 )^ reported that HFNC was associated with a larger number of ventilator-free days than helmet-based NIV, indicating improved outcomes and potentially lower mortality rates.

The observed survival advantage may be attributed to the ability of HFNC to provide more effective oxygenation and ventilation support, improving respiratory function and reducing the risk of complications and mortality. HFNC delivers a higher flow rate and FiO_2_, mitigating the severity of respiratory failure and enhancing patient outcomes.^( 11 , 14 , 16 )^

Several studies^( 11 - 17 , 18 )^ had similar protocols, with HFNC flow rates ranging from 30 to 60L/min and FiO_2_ titrated from 0.4 to 1.0, targeting a peripheral oxygen saturation higher than or equal to 93%. Mauri et al.^( 24 )^ reported that HFNC improves oxygenation by increasing airway pressure, end-expiratory lung volume, and carbon dioxide clearance, thereby alleviating hypoxemia in patients with mild to moderate acute respiratory failure.

In our meta-analysis, HFNC was superior to NIV in terms of the mortality rate (OR=0.62, 95%CI=0.51-0.76, p<0.0001). This contrasts with the findings of He et al.,^( 6 )^ who reported no significant effect (OR=1.41, 95%CI=0.72-2.74, p=0.31). However, their analysis did identify a lower 28-day hospital mortality associated with HFNC (OR=1.81, 95%CI=1.12-2.92, p=0.02). Similar results have been revealed in other meta-analyses, including those by Peng et al.^( 21 )^(OR=0.66, 95%CI=0.51-0.84, p<0.001) and Beran et al.^( 25 )^ (OR=0.81, 95%CI=0.66-0.98, p=0.03).

A similar association between NIV and mortality, regardless of the etiology of acute respiratory failure, was previously observed. Thille et al.^( 26 )^ conducted an observational study on patients with acute respiratory failure unrelated to COVID-19, reporting a 50% failure rate with NIV, which was associated with increased mortality. Despite sample heterogeneity, these findings suggest that NIV failure remains a challenge in predicting which individuals are likely to benefit from this therapy.

Conflicting results regarding the association between non-invasive respiratory support and mortality have been revealed in several other studies.^( 6 , 21 , 25 )^ These discrepancies may be attributed to differences in study methodologies, patient characteristics, and underlying comorbidities.^( 24 )^

An important consideration is the distinct therapeutic management, patient compliance, and clinical effects of HFNC and NIV. High-flow nasal cannula is an open system that generates a modest increase in end-expiratory lung volume despite variations in inspiratory flow and mouth opening. This mechanism reduces dead space and improves oxygenation. In contrast, NIV generates positive end-expiratory pressure, which increases functional residual capacity and reduces pulmonary shunt, ultimately lowering the required to breathe and enhancing respiratory function.^( 6 , 16 , 27 , 28 )^

However, NIV is associated with a higher risk of therapy failure due to complications such as skin breakdown at mask contact sites and patient intolerance, which may result in therapy-related anxiety or phobia. HFNC is often recommended for high-risk patients owing to its better compliance rate, physiological adaptation, and overall tolerability despite its lower performance in terms of the PaO_2_/FiO_2_ ratio.^( 29 , 30 )^

In the meta-analysis of the mortality rate, we included six studies^( 12 , 14 , 17 - 20 )^comprising 1,897 participants for HFNC (827) *versus* conventional oxygen therapy (1,069), and an additional four studies^( 15 , 18 - 20 )^ comparing NIV (707 participants) with oxygen therapy (1,257 participants). The findings demonstrated no difference between conventional oxygen therapy and HFNC (OR=1.06, 95%CI=0.84-1.33; p=0.64), whereas NIV was associated with lower mortality rates than oxygen therapy (OR=1.59, 95%CI=1.26-2.01, p<0.0001). However, we acknowledge that patients receiving conventional oxygen therapy might have had less severe respiratory failure at the time of therapy initiation than those treated with HFNC or NIV. As oxygen therapy is frequently administered earlier in the disease course when symptoms are milder, this might have partially explained the lower baseline mortality risk observed in such patients.^( 13 )^ Furthermore, high heterogeneity was observed in this analysis, primarily influenced by two cohort studies that carried substantial weight in the meta-analyses.^( 15 , 20 )^

In another meta-analysis,^( 31 )^ HFNC was associated with a lower mortality rate than conventional oxygen therapy (OR=0.54 [95%CI=0.30-0.97, p=0.04], χ^2^=21.57, I^2^=77%). These discrepancies may be explained by variations in study designs across the studies included in the analyses. Importantly, although the referenced studies and the meta-analysis provide insights into the comparative effectiveness of these interventions, individual patient characteristics, disease severity, and resource availability should be considered when treatment decisions are made.

Our study contributes valuable insights into the comparative effectiveness of these respiratory support strategies; nonetheless, several limitations should be acknowledged. These include the RCTs and the wide variability in patient clinical profiles, which might have introduced bias and increased heterogeneity in the results, respectively. Our results provide a foundation for informed clinical decision-making in future respiratory disease outbreaks and highlight the need for further research to optimize the management of acute respiratory failure induced by COVID-19.

## CONCLUSION

This meta-analysis indicates that, in patients with COVID-19-related acute respiratory failure, high-flow nasal cannula therapy and non-invasive ventilation result in comparable intubation rates, while high-flow nasal cannula therapy is associated with lower mortality. These findings strengthen the evidence supporting high-flow nasal cannula therapy as a safe and effective first-line noninvasive respiratory support strategy in this population. The results also highlight the need for structured clinical protocols and adequate monitoring to guide the selection and timely escalation of noninvasive respiratory support.

## SUPPLEMENTARY MATERIAL

Jakson Henrique Silva, Anna Luísa Araújo Brito, Redha Taiar, Bruno Amorim Moraes, Anderson Brasil Xavier, Wagner Souza Leite, Maria das Graças Rodrigues de Araújo, Daniella Cunha Brandão, Armele de Fátima Dornelas Andrade, Shirley Lima Campos

DATA AVAILABILITY:

The underlying content is contained within the manuscript.
